# Handling natural hazards in Indonesia amid the COVID-19 pandemic: Muhammadiyah’s response and strategy

**DOI:** 10.4102/jamba.v14i1.1254

**Published:** 2022-04-28

**Authors:** Muchammad Ichsan

**Affiliations:** 1 Magister of Law, Muhammadiyah University of Yogyakarta, Yogyakarta, Indonesia

**Keywords:** natural hazards, COVID-19, response, strategy, Muhammadiyah, Indonesia

## Abstract

Indonesia is prone to natural hazards, which have continued to occur even during the coronavirus disease 2019 (COVID-19) pandemic. Therefore, this study explored the response and strategy employed by Muhammadiyah, one of Indonesia’s moderate Islamic organisations, in dealing with natural hazards during this pandemic. A qualitative descriptive method was used in this study, and the data collection procedure involved finding related literature, reports, and decrees. Online interviews were also conducted with the Muhammadiyah Disaster Management Center (MDMC) administration to strengthen the data. Subsequently, this study discovered that Muhammadiyah responded by aiding victims of natural hazards, which occurred in various regions in Indonesia during the COVID-19 pandemic. The strategies employed comprise Muhammadiyah COVID-19 Command Center (MCCC) to handle COVID-19, alongside essential recommendations to the MDMC network throughout Indonesia and the various arms of the government for dealing with natural hazards during the pandemic. Also, it showed commitment to handling these hazards by establishing a standard operating procedure for Muhammadiyah volunteers and represented Indonesia during a presentation at the World Health Organization.

## Introduction

Indonesia is prone to natural hazards (Djalante 2018), which is expected, as geology shows that the country is at the confluence of three active tectonic plates, namely the Indo-Australia, Eurasia, and Pacific plates in the south, north, and east, respectively (Saputra 2018). These three plates move and collide with each other, causing earthquakes, volcanic paths, and faults that threaten the territory. These hazards are coupled with environmental damage and uncontrolled utilisation of natural resources, and their frequency and the accompanying rate of damage and fatalities are increasing (Robi Amri 2011). Natural hazards occur regardless of the situation, as these events hit many areas in Indonesia, even during the coronavirus disease 2019 (COVID-19) pandemic (Nabila 2021; Ramadhan 2021). According to the National Disaster Management Agency (BNPB), there has been 1205 natural hazards from January 1 to April 30, 2021, which were dominated by hydrometeorological events, such as floods, tornadoes, and landslides. Flood hazards were the most frequent, with 501 incidents, followed by 339 tornadoes, 233 landslides, alongside 97 forest and land fires, 18 earthquakes, 16 tidal and abrasion waves, and one drought. Judging from that period, the number of natural hazards increased by 1% from the previous year, as did the death toll by 1.83%. These hazards left 479 dead, 60 missing, 12 900 injured, and 5 million displaced (Jati 2021).

Before the COVID-19 pandemic erupted in Indonesia in early 2020, studies about Muhammadiyah and its relation to hazards solely examined their role during these events. However, since the outbreak of the pandemic, many studies have focused on the organisation’s role in dealing with the pandemic from various aspects, such as health, education, social, and religious guidance. Falahuddin (2020) found that Muhammadiyah has contributed to the handling of COVID-19 by forming a task force called Muhammadiyah COVID-19 Command Center (MCCC), the vanguard in COVID-19 prevention in the country. In addition, the organisation supports the health protocols and policies implemented to prevent the transmission of COVID-19, especially physical distancing practices. Meanwhile, Ichsan (2020) examined Muhammadiyah’s position in Islamic philanthropy and its contribution to overcoming the pandemic through health, education, social and religious guidance. Suyadi, Nuryana and Fauzi (2020) wrote about the reasoning of Disaster *Fiqh* [deep understanding], namely the Muhammadiyah product document for answering contemporary problems, mainly geological and non-geological hazards. This subsequently became the normative basis for alleviating health hazards and has been actualised in the mitigation of COVID-19, medical health movements, the reconstruction of *fiqh* worship during the emergency, and handling hazards theologically.

Consequently, this study supplemented the limited scope of previous research, which precluded the occurrence of natural hazards during the COVID-19 pandemic from their investigations. The problem of this study is how is Muhammadiyah’s response and strategy in dealing with natural disasters during the COVID-19 pandemic? Therefore, this study aims to explore Muhammadiyah’s response and strategy for handling natural hazards in various regions of Indonesia during this pandemic. Natural hazards have continued to afflict numerous areas throughout the COVID-19 pandemic, thereby requiring the disclosure of Muhammadiyah’s response and strategy for dealing with these incidents during this period.

The basis of this study is the argument that besides endeavouring to overcome the continued spread of COVID-19 in Indonesia, Muhammadiyah is not silent about the natural hazards occurring in various regions. The incidence of natural hazards during a pandemic will undoubtedly make people more miserable and helpless. Therefore, Muhammadiyah must remain committed to performing this humanitarian task, although the difficulties and obstacles are extraordinary than regular times without a pandemic. This study explored the response and strategy used by this organisation in handling natural hazards during this period and proceeded to establish Muhammadiyah’s humanitarian contributions and lay the groundwork for stakeholders to make future policy decisions.

## Literature review

Several researches have been conducted worldwide to examine the link between the COVID-19 pandemic and natural hazards. Hariri-Ardebili and Lall (2021) precisely accomplished that. They shed light on the reciprocal relationship and interplay between a natural disaster and a pandemic breakout, as well as the risk to human life. Hariri-Ardebili (2020) also looked at three potentially dangerous occurrences through the prism of a pandemic. He created high-risk, low-probability scenarios by combining a pandemic with a natural hazard or a severe emergency. He also looked at the qualitative effects of these multi-hazard situations. Meanwhile, Chingombe and Musarandega (2021) looked at how the survivors of the Cyclone Idai disaster in Eastern Chimanimani, Zimbabwe, were self-cultivated and eventually used social capital to confront the new socio-economic problems posed by the COVID-19 pandemic. In addition, Silva and Paul (2021) studied the potential impact of earthquakes during the 2020 COVID-19 pandemic. They concluded that the global spread of the COVID-19 virus poses unprecedented challenges to disaster risk management, particularly in adopting or developing reaction and readiness plans for simultaneous natural hazards.

Previous research about Muhammadiyah and its relation to natural hazards in Indonesia explored the subject from two aspects, before and after the COVID-19 pandemic. Before the outbreak of the pandemic, the research examined Muhammadiyah’s role in handling natural hazards in various regions of Indonesia. Then, the focus became dealing with the disease from various angles such as health, education, social, and religion, after the pandemic hit the country in March 2020. The following section presents Muhammadiyah’s concept about disasters and the COVID-19 pandemic and studies on Muhammadiyah’s attitude towards natural hazards before and after the COVID-19 pandemic.

### Muhammadiyah’s concept about disasters and the COVID-19 pandemic

Muhammadiyah defines a disaster as:

[*S*]erious disturbances caused by natural and human factors, which can paralyze the functions of a society built to sustain survival, protect assets, preserve the environment and ensure human dignity, as part of religious orders. The paralysis of this function is due to widespread human, material, economic, or environmental losses that go beyond the ability of affected communities to cope by using their resources. (Muhammadiyah 2015, p. 11)

The problem of disaster management is not foreign to Muhammadiyah. Since its establishment in 1912 in Yogyakarta, Muhammadiyah has made tremendous efforts to alleviate the misery and suffering of the people of Indonesia. KH Ahmad Dahlan founded Muhammadiyah to help the people in their suffering (Fauzia 2017). At that time, what KH Ahmad Dahlan and his students did in the field of health was to establish hospitals and clinics, and in the social field to provide child compensation, build poorhouses and provide assistance to the victims of the natural hazard of Mount Merapi (Muchammad Ichsan 2021). In 2007, Muhammadiyah established the ‘Disaster Management Center’. This centre was confirmed to be an institution tasked with coordinating Muhammadiyah resources in disaster management activities by the Muhammadiyah Central Leadership after the 2010 Conference under the name of Muhammadiyah Central Disaster Management Agency. This institution has the English designation ‘Muhammadiyah Disaster Management Center’, abbreviated as MDMC (MDMC 2020).

The way of looking at an event is essential because it will affect the attitude and actions to be taken in the face of the event. Similarly, with the problem of disasters, the way Muhammadiyah views disasters will significantly determine the attitude and actions that will be taken. Here is how Muhammadiyah views disasters (Muhammadiyah 2015): (1) According to Muhammadiyah, a disaster is a form of God’s love for his servants. God’s love for humans can be in the form of grace in various kinds and types, such as sustenance, health, wealth, and security. God’s love can also be things that feel bad or disturbing such as disasters, hazards, and others. Moreover, disaster is, in fact, a test or trial of human faith and deeds. God will test humans first if God wants to elevate them to a higher level and wash away their faults and sins. Disaster is not a form of God’s anger and hatred towards his servants, but rather it is a form of God’s goodness and love for humans, for God will bestow his forgiveness, mercy, and guidance with such a disaster. (2) Muhammadiyah views disaster as the destiny or the provisions and decrees of God that have occurred. Only God knows when and how a disaster will occur, while humans can only know it after it has happened. (3) Disaster as a medium of self-introspection. To be better, humans must always be self-introspective. If a disaster occurs, man reflects on themselves; whatever was done to cause the disaster, man takes it upon themselves. Meanwhile, Muhammadiyah’s attitude towards disasters is as follows (Muhammadiyah 2015): (1) All parties associated with disasters, such as individuals, families, communities, and governments, must have a complete awareness of disasters and have a positive attitude towards them. The purpose of a positive attitude towards disasters here is a responsible attitude to handle disasters so that there is no mutual responsibility in the event of a disaster. (2) Disasters often do not affect a particular group of people, but affect others around them; even the scope of disasters can reach a large area. Therefore, all parties are responsible and must help each other to overcome it. (3) The aim of the spirit and mutual help is to fulfil the rights of disaster victims. When a disaster strikes, the victims have rights that must be fulfilled. These rights include the right to manage disaster risk, the right to manage vulnerabilities, emergency assistance, rehabilitation and reconstruction, the right to implement disaster management systems, and the right to be resilient. In addition to these rights, it is necessary to add here the religious rights of the community and related rights. (4) All parties must comfort disaster victims so that they do not ‘feel alone’ and despair in the face of disasters, because there are still many people who care for them and there is hope to rebuild what has been damaged or lost due to disaster. In handling the COVID-19 pandemic, Muhammadiyah refers to the World Health Organization and the Indonesian government. The World Health Organization declared the COVID-19 outbreak a pandemic that became a global problem (World Health Organization 2020), and the Government of Indonesia, through the Task Force to Accelerate the Handling of COVID-19, declared the COVID-19 outbreak a non-natural hazard (Covid-19 2020). Considering the rapid spread of COVID-19, Muhammadiyah declared the outbreak as an extraordinary event that must be immediately dealt with well-coordinated efforts. Muhammadiyah encouraged the government to involve all parties to cooperate and synergise with socialisation measures and policies that are open and comprehensive (Muhammadiyah 2020b). Muhammadiyah itself has consistently handled the COVID-19 pandemic (MDMC 2020). Among other things, by forming MCCC, a unique team was formed to deal with COVID-19 (Muhammadiyah 2020a). Since its founding, the team has been working and handling the pandemic situation. Among the results of its work, assistance in health is needed, such as providing hospitals, doctors, medical personnel, and medicines. Assistance in education includes creating educational media, subsidising credit for students, and discounting student study fees. Assistance in the social sector includes cash assistance and distribution of foodstuffs. In addition, through the Tarjih and Tajdid Assembly, Muhammadiyah provides religious guidance for its members during this pandemic to avoid being confused about performing worship (Ichsan 2020).

### Muhammadiyah and natural hazard management

The study of Muhammadiyah’s role in handling natural hazards began several years ago. Baidhawy (2015) explored the organisation’s theological stand on disasters, its role, disaster management approaches, and mitigation strategies. The study pictured several ways to involve others and partner with multiple national and international stakeholders. Also, Mustika Kurniatri and Sunaryadi (2016) investigated the method, Quality Function Deployment (QFD), used in improving the quality of disaster-related services in PKU Muhammadiyah Bantul Hospital by formulating management efforts based on the wishes of patients and disaster victims. Then, Hilman (2018) found that the spirit of Al-Maun has caused the MDMC to be colourful and exhibit a distinguishing characteristic in conducting disaster management activities. This is depicted by the support in helping those in need, which is realised through a partnership with autonomous Muhammadiyah organisations or other parties, such as the government or private institutions. Muhammadiyah Disaster Management Center achieves this objective through disaster management activities such as victim identification, reconstruction, rehabilitation, and recovery. Furthermore, Sakban, Maemunah and Hafsah (2020) found that MDMC adequately implemented disaster management education for volunteers in West Nusa Tenggara. The centre synergises with the government in rehabilitating and mitigating earthquake disasters through structural and non-structural approaches. Muhammadiyah’s involvement in disaster management is not solely domestic, as the organisation raised funds and sent humanitarian teams for other disaster relief and development programmes in neighbouring Southeast Asia countries, such as the Philippines and Thailand, and disaster-affected nations like Nepal and Palestine (West Bank and Gaza) (Latief & Nashir 2020). Following the study by Bush (2015) a few years earlier, Muhammadiyah’s leading role in disaster and humanitarian assistance in Indonesia has led to its inclusion in international political discourses on humanitarian aid. The above studies highlight the organisation’s role in handling natural hazards before the COVID-19 pandemic. Hence, Muhammadiyah never stayed silent during hazards that resulted in many victims, showing that the spirit of Al-Maun, which entails helping the needy, has indoctrinated Muhammadiyah deeply.

### Muhammadiyah’s response to the COVID-19 pandemic

The COVID-19 pandemic began to spread in Indonesia in March 2020, and studies on Muhammadiyah’s role in its management were conducted a few months later. These studies are increasing, as they are conducted for various concerns, including general and mental health, social, religious, educational, economic, and other matters. For example, Djalante et al. (2020) explored the health aspect and reported that Muhammadiyah formed the COVID-19 Command Center and allocated hospitals. The organisation has been transformed into one of the most agile health- and hospital-based emergency responses promoters. Meanwhile, MDMC has been instrumental in the disaster response system in Indonesia, in which the concerned institutions provide clinical services to COVID-19 patients in their hospitals. In the psychological field, Syamsurijal and Sarwan (2021) examined the psychological conditions of elementary school teacher-education students (PGSD) of Muhammadiyah Buton University in implementing online learning during the pandemic. They discovered that the students experienced psychological problems following the online learning programmes through psychological indicators.

Additionally, Wicaksana Prakasa et al. (2021) explored the social field by examining Muhammadiyah’s role as a civil society entity for guarding the distribution of social assistance throughout the East Java province. They found that the Participatory Action Research conducted by the Muhammadiyah residents in this area is a practical step and an excellent example of suitable structural and cultural approaches that various entities can use to mitigate the potential for social assistance corruption during the pandemic. Meanwhile, Lestari, Hamsia and Setiyawan (2021) analysed the adaptation strategies employed by two Muhammadiyah inclusion schools in Surabaya, Indonesia, namely Sekolah Peduli Anak Hebat (SPAH) and Sekolah Kreatif Surabaya, in the education sphere. The study discovered that these strategies could help coordinate parents, teachers and students, build community cooperation, and make flexible and accessible learning policies. In the business field, Hanif, Fery and Raheni (2020) discovered the roles of Muhammadiyah volunteers in assisting micro-businesses in the central Sulawesi province to survive the COVID-19 pandemic, for example, the provision of a capital injection of 4 million rupiahs, had a very positive effect. Also, Isngadi and Khakim (2021) discovered numerous issues that need to be evaluated in the law’s implementation after examining the effectiveness of applying Law No. 24 of 2007 and the Muhammadiyah Disaster *Fiqh* in dealing with COVID-19 through law and religion. The above research shows that investigations on Muhammadiyah’s response and strategy in handling natural hazards during the COVID-19 pandemic are still very limited or unexecuted, thereby creating a need for a study on this subject as a basis for future action by stakeholders.

## Methods

This study employed qualitative descriptive methods to describe Muhammadiyah’s response and strategy in handling natural hazards in Indonesia during the COVID-19 pandemic. These incidents occurred in many areas between January 2020 and June 2021, and Muhammadiyah did not stay silent, as the organisation dealt with these hazards during the COVID-19 outbreak.

The data were collected from related literature, reports, and decrees that support the analysis of Muhammadiyah’s response and strategy in handling natural hazards during the COVID-19 pandemic and strengthened by online interviews through Microsoft Teams and Zoom. This method was used to comply with social and physical distancing policies that prohibit face-to-face interviews. These interviews were designed to elicit information about the study topic, and the participants included the Chairman, Deputy Chairman, and Secretary of MDMC, who is concurrently the Secretary of MCCC.

Subsequently, the data were analysed according to the stages of qualitative descriptive studies, namely data reduction, display, and conclusion drawing. The reduction involved selecting resources relevant to the study topic and sorting out less supportive information. Then, the data were compiled and described to explain the information and interpreted by providing definitions and meanings before the conclusion was drawn. The innovation and creativity of the interpretation were performed to determine the meaning behind the collected data while ensuring its originality.

### Ethical considerations

Ethical clearance to conduct the study was obtained from the Research and Innovation Institute, University of Yogyakarta, reference number: 167 /LRI-UMY/X/202L.

## Results

Muhammadiyah referred to natural hazards during the COVID-19 pandemic as multi-hazards because of the increased dangers that threatened the victims and volunteers who helped. These persons are highly vulnerable to the threat posed by the ongoing pandemic (Setiawan 2021). Nevertheless, Muhammadiyah has remained steadfast in its mission and undeterred in fulfilling its humanitarian obligation by helping numerous victims of natural hazards that occurred in various regions of Indonesia (Husein 2021). These hazards include the landslide in Bogor, the Seroja storm in East Nusa Tenggara, and floods in South Kalimantan (Kholis 2021). The organisation’s response and strategy in dealing with natural hazards during the COVID-19 pandemic is discussed in the following sections.

### Muhammadiyah’s response in handling natural hazards during the COVID-19 pandemic

**FIGURE 1:**
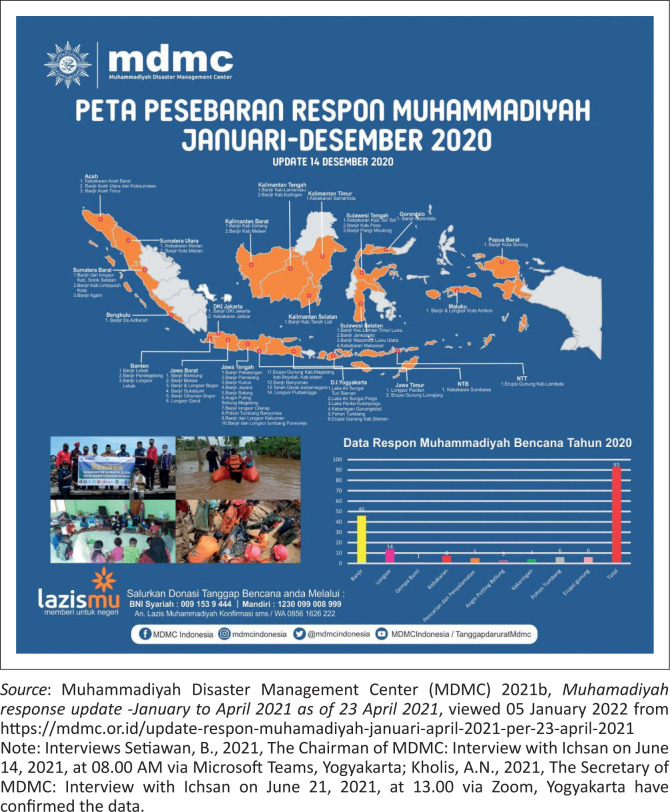
Muhammadiyah response distribution map from January to December 2020.

**FIGURE 2:**
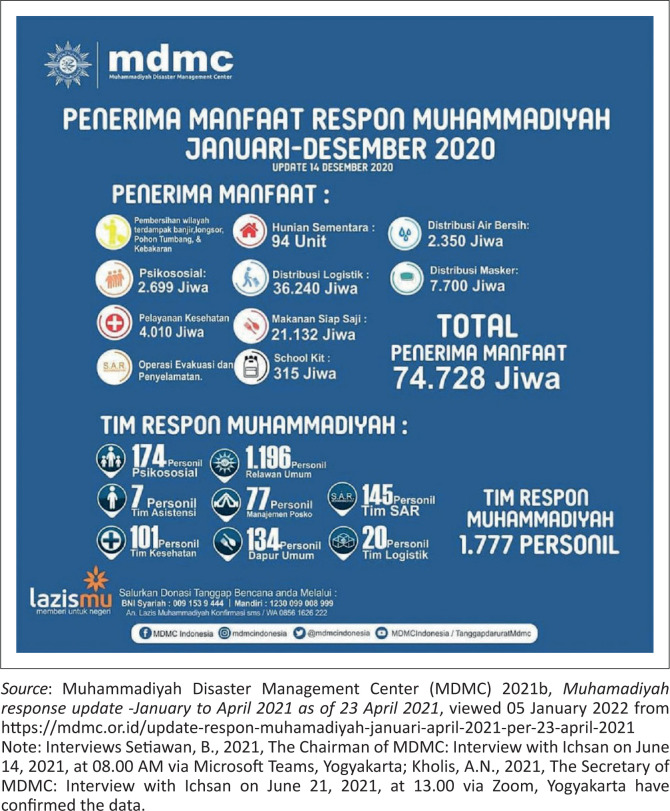
Muhammadiyah response beneficiaries from January to December 2020.

Muhammadiyah continually strives to handle natural hazards, and its efforts have remained consistent despite the COVID-19 pandemic. The organisation responded to these incidents by aiding people in many areas affected by natural hazards from January 2020 to June 2021, as revealed by the two sets of data below:

Firstly, the data on its response to natural hazards between January and December 2020 show that:

- The Tanggap Darurat, Rehabilitasi dan Rekonstruksi [Emergency Response, Rehabilitation, and Reconstruction] Division (TDRR) of the MDMC of the Muhammadiyah Central Leadership have conducted 103 hazard responses, involving 49 floods, 14 landslides, one earthquake, 9 fires, 7 search and rescues, 6 tornadoes, 5 droughts, 6 fallen trees, and 6 mountain eruptions.
- There have been three responses in Aceh, West Sumatra, Central Sulawesi, and Banten each, two each in North Sumatra, West and Central Kalimantan, alongside DKI Jakarta, East Java, and Nusa Tenggara Barat (NBT). Also, there have been one each in South Sumatra, Bengkulu, Lampung, South and East Kalimantan, Gorontalo, Maluku, West Papua, and Nusa Tenggara Timur, alongside four South Sulawesi, 10 West Java, 16 Central Java, and 12 DIYogyakarta responses.
- Total expenditure was Rp 7 552 887 757.
- There were 1965 Muhammadiyah Response Team members, consisting of 174 psychosocial personnel, 1234 general volunteers, alongside seven assistance teams, 77 post-management, 116 health team, 145 available kitchen, 175 SAR team, and 37 logistic team personnel.
- Total beneficiaries include 86 200 people, comprising the cleanup of areas affected by floods, landslides, fallen trees, and fires. There were 94 temporary housing units, 3200 recipients of clean water, 2753 psychosocial assistances, 42 888 logistics distributions, 7700 mask receivers, 4010 healthcare beneficiaries, 25 052 ready meals, and evacuation and rescue operations, and 315 school kits. All of the above data is included in Figure 1 and Figure 2.

**FIGURE 3:**
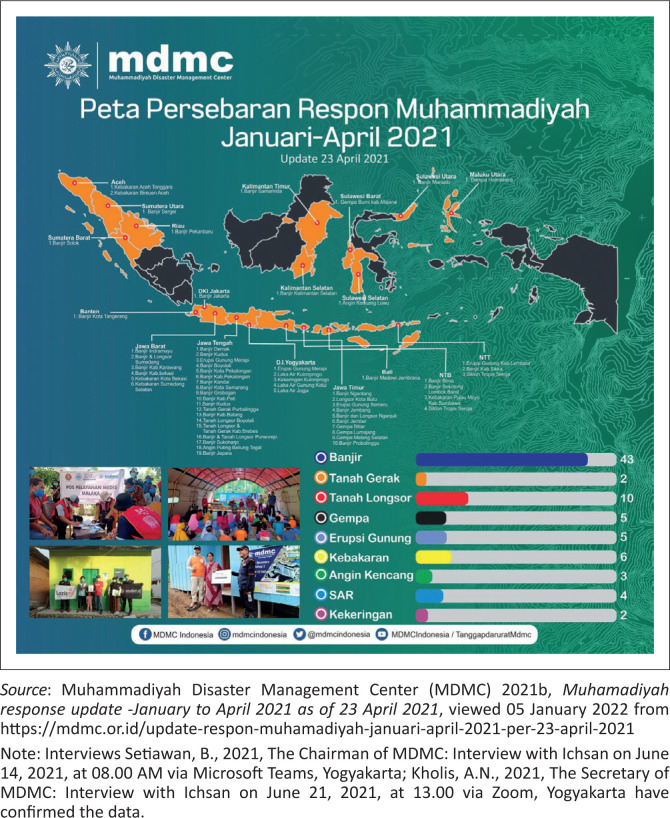
Muhammadiyah response distribution map from January to April 2021.

**FIGURE 4:**
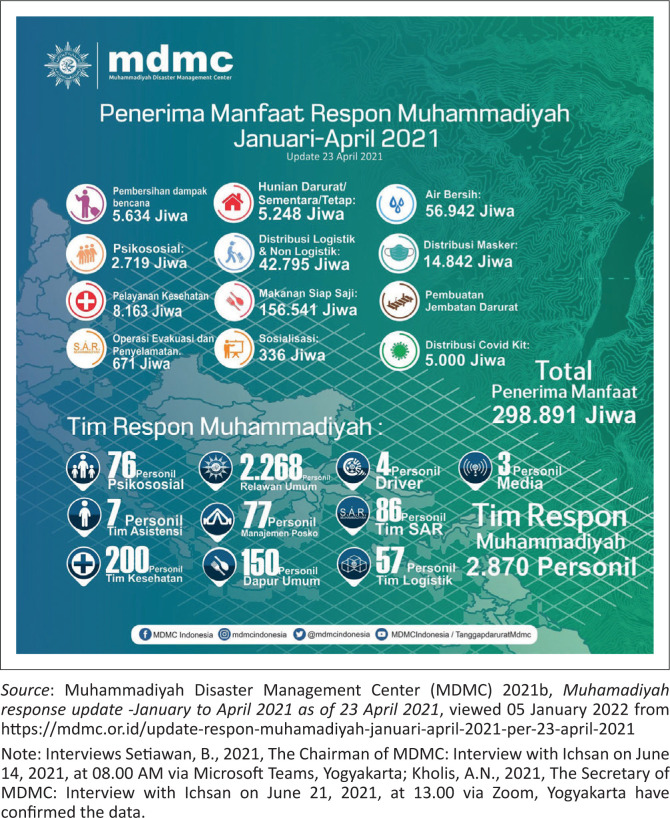
Muhammadiyah response beneficiaries from January to April 2021

**FIGURE 5:**
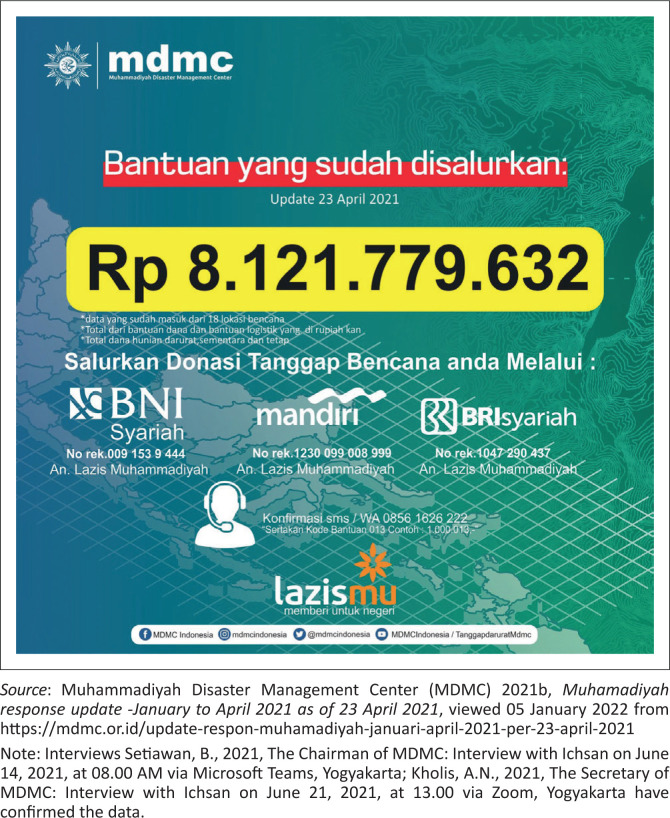
Aid that has been distributed on 23 April 2021.

Secondly, the data concerning Muhammadiyah’s response to natural hazards between January – April 2021 showed that:

- TDRR of MDMC of Muhammadiyah Central Leadership implemented 80 hazard responses from January to April 23, 2021.
- Central Java had the most responses of 19, followed by East Java, West, Yogyakarta, and NTB, at 10, 6, 5, and 4 times. The rest were in 14 other provinces, which occurred below three times in each region.
- Of the total 80 responses that were implemented, MDMC distributed Rp. 8 121 779 632. These funds came from donations from various parties, including extended Muhammadiyah members and the public channeled through Lazismu, and assistance from MDMC partner agencies, such as Swiss Solitaire, CRS, and Allianz.
- There were 298 891 beneficiaries of MDMC funds through various response activities, namely SAR operations, ready-to-eat food distribution, COVID-19 kits, clean water, logistics and non-logistics, psychosocial assistance, health services, environmental cleaning, emergency, temporary or permanent shelters, socialisation, and emergency bridges.
- There were 2870 volunteers, comprising about 2268 general, 200 medical, 76 psychosocial, 7 assistants, 150 available kitchen, 86 SAR team, 57 logistics, 3 media personnel, and 4 drivers.
- In April 2021, Muhammadiyah volunteers plunged into three hazard areas, namely West Nusa Tenggara, particularly Bima and Dompu due to extreme rainfall, East Nusa Tenggara because of the cyclone Seroja, and East Java due to the earthquake that hit the southern regions, such as Blitar, Lumajang, Malang, and Jember.
- In West Nusa Tenggara, the local volunteers helped flood-affected residents in Bima and Dompu by performing an intensive environmental cleanup, logistical assistance, ready meals, and accessible health services.
- After the landslide in East Nusa Tenggara, the MDMC Head sent National Muhammadiyah Emergency Medical Team (EMT) to support the local volunteers. The services conducted include free medical treatment, logistical and psychosocial assistance, and improved clean water advice for the residents.
- The volunteers in Blitar, Lumajang, Malang, and Jember of East Java performed various activities to help the survivors, such as distributing logistics and ready meals, cleaning earthquake debris, psychosocial assistance, and free medical examinations. All of the above data is included in Figure 3, Figure 4 and Figure 5.

### Muhammadiyah’s strategy in dealing with natural hazards during the COVID-19 pandemic

**FIGURE 6:**
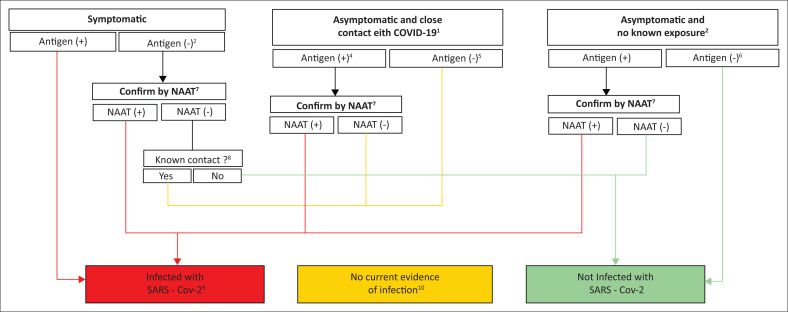
Standard operational procedure for the assignment of Muhammadiyah volunteers during the COVID-19 pandemic.

Muhammadiyah employs various strategies in handling natural hazards during general or everyday circumstances. However, new strategies were formulated during the COVID-19 pandemic to suit the situation and conditions, including:

- The Muhammadiyah Central Leadership formed the MCCC on March 14, 2020, based on the Decree of Muhammadiyah Central Leadership Number 2825/KEP/I.0/D/2020 concerning the Establishment of MCCC in charge of coordinating various COVID-19 pandemic prevention programmes involving Muhammadiyah potential and networks (Muhammadiyah 2020).
- The provision of recommendations on handling natural hazards during the pandemic to the entire MDMC network throughout Indonesia and various arms of the government (MDMC 2021a). The recommendations are listed in Table 1.
- Muhammadiyah also set a Standard Operational Procedure (SOP) for volunteers to maintain their health as well as that of the victims of natural hazards during the pandemic (Muhammadiyah 2021a), which is shown in Table 2 and Figure 6.
- The EMT of the Muhammadiyah Deployment during the COVID-19 pandemic represented Indonesia and made a presentation at the World Health Organization (WHO) programme, hosted virtually in Geneva, Switzerland on Thursday 29 April 2021 (Muhammadiyah 2021b; Nihayati 2021).

**TABLE 1:** Muhammadiyah Disaster Management Center (MDMC) Recommendations on Handling Natural Hazards During the Pandemic.

Variable	Description
**Recommendations for the entire MDMC network throughout Indonesia**	A shared commitment to managing the shift in disaster management focus from centralised emergency response to community-based risk reduction. This activity gained momentum during the COVID-19 pandemic and involved using volunteers, facilitators, and interregional assistance personnel as a last resort for risk management and the principle of *hifdzun an-nafs* or putting first things first. Strengthened disaster risk reduction systems in families, communities, and among worshippers under the Disaster Resilient Society (MASTANA), Disaster Safe Education Unit (SPAB), and Disaster Safe Hospital (RSAB) principles. This has been a commitment of Muhammadiyah for the last 10–15 years and must be executed by adapting to pandemic conditions where health protocols cannot be enforced. Strengthened emergency preparedness and disaster response by the Muhammadiyah network throughout Indonesia. This is done by enhancing the safety management of volunteers, programme implementers, and citizens affected by COVID-19 conditions, which require the enforcement of health protocols, prioritise the handling of emergency response by local volunteers, and are involved in cluster/sector coordination mechanisms. Strengthened network of organisations, volunteers, facilitators, and partnership cooperation to enhance the disaster risk reduction system and community-based emergency response process, adapted to the pandemic conditions. Additionally, it participates actively in the Forum on Disaster Risk Reduction in Cities and Districts. To exemplify the implementation of COVID-19 health protocols and strengthen the Muhammadiyah COVID-19 Command Center (MCCC) at all levels of the community leadership. To be a strategic partner and implementer for the central and regional governments in the synergy of COVID-19 countermeasures.
**Recommendations for the Central and Local Governments**	The prioritisation of policies and budgets for implementing community-based disaster risk reduction principles to ensure the resilience of the minor community groups in handling all disaster threats, as inter-regional mobility for volunteers and disaster relief must be suppressed during the pandemic. To strengthen the community’s multi-hazard disaster emergency response system to support strong communication, logistics, and supervision systems following COVID-19 health protocols. To strengthen cooperation between the arms of government, community organisations, and non-governmental organisations to be more effective in handling natural hazards and the pandemic, based on the strength of the community. There must be data or evidence, along with expert opinions (scientific-based approach), in formulating government policies and strategies for handling pandemics by putting the safety of the people above other interests. Every policy prepared in response to natural hazards and the COVID-19 pandemic must be sensitive to women and vulnerable groups. To ensure prioritised efforts to protect the safety of volunteers and affected citizens in emergency response activities and natural hazard recovery during the pandemic; Personal Protective Equipment (PPE), tracing and testing devices, and isolation infrastructure for confirmed COVID-19 cases must be provided.

*Source:* Muhammadiyah Disaster Management Center (MDMC), 2021a, *Penguatan Sistem Penanggulangan Bencana dalam Masa Pandemi Covid-19,* Yogyakarta, viewed from file:///C:/Users/umy/Downloads/Rekomendasi Rakerpim MDMC Penguatan Sistem Penanggulangan Bencana dalam Masa Pandemi Covid-19.pdf

Note: Setiawan (2021) and Kholis (2021) confirmed the data.

**TABLE 2:** Standard operational procedure for the assignment of Muhammadiyah volunteers during the COVID-19 pandemic.

Variable	Description
**A. General Requirements**	Volunteer Criteria: ≤ 60 years old. No comorbidity. Have a negative PCR antigen or rapid swab test (valid for 2 ´ 24 h). Not undergo therapy for certain diseases. Not pregnant or breastfeeding. A volunteer exposed to COVID-19 is allowed to serve, providing the PCR swab results showing negative results and/or the individual is declared fit from the Doctor in Charge* (considering there are long COVID-19 cases and persistent positive PCR). Sign an informed consent form indicating their willingness to adhere to quarantine/isolation procedures if necessary. Willing to work in the MDMC coordination. Administrative Needs: Each agency’s letter of duty (1 soft copy, five hard copies). Medical logistics pass (1 soft copy, five hard copies). Rapid antigen test printout (1 soft copy, five hard copies). Copy of STR (1 soft copy, five hard copies). Copy of SIP (1 soft copy, five hard copies). Have an identity card and/or driver’s license (1 soft copy, five hard copies). Volunteer Personal Equipment: Field shirts (quick-dry shirts and field pants). Field shoes and sandals. Hat. Enough change of clothes Personal medicine. Raincoat. Rain cover. Small bag for identity. Personal drinking tableware. Operational funds for personal expenses during the duration of the assignment. Stationery. A laptop (1 only per team). Flashlight/headlamp. Solar power bank and electric power. Prayer equipment. Disposable gown for volunteers with a minimum assignment of 7 days or according to the assignment period (medical volunteers only). PPE coverall with sponge-bound material for volunteers with a minimum assignment of 7 days or according to the assignment period (especially for medical volunteers). Mask: At least 4 times a three-layer cloth mask (assignment period + travel). For example, the assignment period of 7 days + 2 days of travel, then bring a cloth mask as much as 4 ´ 3.5 = 16 pieces. Surgical masks with a required amount of 3 times the assignment period + length of travel (e.g. assignment period 7 days + 2 days trip then at least bring 27 surgical masks) or N95/KN95 mask (medical volunteers only); 3 times 1/2 (assignment period + length of travel). For example, the assignment period is 7 days + 2 days trip, then at least bring 2 ´ 3.5 = 8 masks N95 / KN95. Hand sanitiser; 100cc minimum. 70% alcohol content; 100cc minimum. Face shield (medical volunteers only). Goggles (medical volunteer only). Follow the team leader’s and TDRR coordinator’s briefing/directive.
**B. SOP Volunteers During Assignment**	Follow the directives of the Poskor and Posyan chairmen, and the team leader. Health protocols during assignment: Always use the mask properly (covering nose and mouth). Using masks during service: N95/KN95 and the face shield for medical volunteers. Surgical masks for non-medical volunteers. Changing the mask regularly, based on the type of mask used, namely: For cloth masks, every 6 h, replace the mask with a clean one. The mask can be soaked with detergent for at least 20 min and dried/ironed. Replace surgical masks with a new one every 8 h. Old masks should be dumped in particular places for medical waste. Replace N95/KN95 masks with a new one every 12 h. The old mask can be dried or hung in a location with good ventilation for 2 days. Wash the hands as often as possible, especially after touching the logistics materials of other teams. Avoid eating with other volunteers, especially in enclosed places, where a distance of > 2 meters cannot be maintained. Take a shower when returning to the post and change to clean clothes. Change the mask after every daily task. Ensure room’s ventilation is in good condition (free air in and out). If the room is air-conditioned, there must be a water purifier with a HEPA 13 filter. Set a maximum of 1 h for meetings or coordination. Keep at least 1 metre between volunteers and survivors during meetings or gatherings. Dispose of used PPE in infectious trash cans (masks should not be disposed of haphazardly). Until proven otherwise, every survivor is considered COVID-19 positive hence every volunteer must wear the necessary PPE.
**C. SOP Return of Volunteers**	Perform the rapid antigen test 1 day before return for ticket management needs. Perform the rapid antigen test once it arrives at the location of origin (optional). Self-quarantine for 6–7 days. Perform the rapid antigen test at the end of the quarantine period for finalisation to ensure the individual was not infected during travel and assignment.
**D. SOP Close Contact**	The definition of close contact is a person with a history of contact with probable or confirmed COVID-19 cases. The contact history includes: Face-to-face contact with probable or confirmed cases within a 1-metre radius for at least 15 min. Physical contact with suspected or confirmed cases, such as shaking or holding hands. People who provided immediate treatment of probable or confirmed cases without using standard PPE. Other circumstances indicating contact, as determined by the local medical team’s risk assessments (e.g. eating together, sleeping close without wearing masks). Volunteers who meet the criteria of close contact: Immediately report to the team leader and the Posyan head. Self-isolate for at least 7 days (the agency’s responsibility is to send volunteers). Conduct rapid antigen tests on the 5th day after contact or the 7th day of self-isolation (the agency’s responsibility that sent volunteers). Maintain body condition, diet/drink, and take supplements. Report to the team leader and the Poskor chairman if there are any symptoms or complaints.
**E. SOP Health Protocol in POSKOR (Coordination Post) or POSYAN (Service Post)**	Ensure all local and outside the area volunteers, including air, sea, and land routes, have conducted rapid antigen and/or swab PCR tests by requesting a hard copy of the results. For volunteers who have not performed the rapid antigen or PCR swab test: Either of these tests should be conducted on-site (where possible). If unable to perform these tests, return to the original location. For negative results, the volunteers should be allowed to continue the assignment. For positive results, the volunteers must quarantine, depending on the clinical conditions experienced. Provide volunteer clinics with crucial tasks: Conduct regular daily health checks to all volunteers on duty at least once a day. Observe the condition of each volunteer based on regular health check results. Share vitamins daily to all volunteers. Schedule sunbathing together every morning. Clean the shared bathroom regularly every 3 days. Provide an adequate number of bathrooms per the number of volunteers serving in the post. Provide an open space for enterprising Poskor/Posyan that will allow the gathering of many people. Provide a particular infectious trash can for the placement of PPE waste. Provide dispensers for drinking. Conduct regular decontamination of all operational vehicles used by volunteers. Establish a particular quarantine facility in Poskor/Posyan with adequate infrastructure, medications, and human resources for volunteers awaiting or with positive rapid antigen test results. Coordinate or report to the local Health Cluster to determine the appropriate follow-up for their clinical conditions. Provide adequate decontamination places for volunteers, consisting of at least: 1 Sprayer for decontamination liquid. 1 Sprayer for soap. Decontamination fluid. Provide special facilities for managing hazardous waste: Incinerator, or Casting system. Disinfect the surface of each logistic package carried by the team at their arrival or after the daily completion of every service. **Reference** Panduan Penanganan COVID-19 Revisi 5 Kementerian Kesehatan Republik Indonesia. COVID-19 resources and guidelines for labs and laboratory workers. Central for Disease Control and Prevention. 2020. (https://www.cdc.gov/coronavirus/2019-ncov/lab/resources/antigen-tests-guidelines.html, diakses tanggal 23 Januari 2021) Panduan bagi Kontak Erat dalam Buku Panduan Revisi 3 Muhammadiyah COVID-19 Command Center (MCCC).

*Source:* Muhammadiyah Disaster Management Center (MDMC), 2021a, *Penguatan Sistem Penanggulangan Bencana dalam Masa Pandemi Covid-19,* Yogyakarta, viewed from file:///C:/Users/umy/Downloads/Rekomendasi Rakerpim MDMC Penguatan Sistem Penanggulangan Bencana dalam Masa Pandemi Covid-19.pdf

TDRR, Tanggap Darurat; Rehabilitasi dan Rekonstruksi [Emergency Response, Rehabilitation, and Reconstruction]; PCR, Polymerase Chain Reaction; STR, Surat Tanda Regristrasi [Certificate of Registration]; SIP, Surat Izin Praktik [Practice License]; HEPA, High Efficiency Particulate Air; MDMC, Muhammadiyah Disaster Management Center; PPE, Personal Protective Equipment; SOP, Standard Operational Procedure.

Note: Setiawan (2021) and Kholis (2021) have confirmed the data.

## Discussion

This study shows the response of Muhammadiyah to natural hazards that occurred in Indonesia during the COVID-19 pandemic. The response covers many areas in the country involving many volunteers, large fund donations, and numerous beneficiaries. This is expected, as non-governmental organisations, including religious organisations such as Muhammadiyah, play crucial roles due to their spearheaded and fast nature, alongside a broader reach of the community. The reasons are, firstly, religious organisations, including Muhammadiyah, have been existing in the society for a long time, even before the state was formed. Secondly, they have a loyal following based on their willingness to help with their funds, time, energy, and possessions. Thirdly, community organisations have a vast network, prominent members or constituents, and a basis on religious values that are believed and often guided and followed by society. Fourthly, they have substantial, vast, and significant assets, and fifthly, they have connections or access to the central and regional governments and network with other organisations with similar values and goals (Ardianto 2021).

Furthermore, this study highlighted Muhammadiyah’s strategy in handling natural hazards during the COVID-19 pandemic. The first was the formation of MCCC to ensure that issues about the pandemic are handled in a well-coordinated manner. Meanwhile, MDMC, as a previously established institution, permanently remains responsible for managing natural and non-natural hazards in regular times and during the pandemic. It is a sound strategy because disaster risk reduction and the COVID-19 pandemic mitigation teams are more organised than before, and the division of tasks and officers are dependable. Hence, better results are expected.

Muhammadiyah’s recommendations about handling natural hazards during the COVID-19 pandemic were also discovered to be aimed at two parties, namely the MDMC network throughout Indonesia, alongside the central and local governments. The recommendations addressed to the entire MDMC network throughout Indonesia comprised directives that should be adhered to, as they are under the central body. The content is significant because it directs actions for dealing with natural hazards during the pandemic. Similarly, the recommendations to governments express the wish and request that the Muhammadiyah standards be applied for the good of the community, and they are congruent with the content directed to the MDMC network.

The SOP for Muhammadiyah volunteers to be deployed to help natural hazard victims during the pandemic is the right strategy as it protects the volunteers directly and the victims indirectly due to the high infectivity of COVID-19. Hence, the lack of SOP or nonadherence may cause the assistance of natural hazard victims to become tragic, as the volunteers and victims may transfer or contract COVID-19. In addition, the representation of Indonesia at the World Health Organization (WHO) programme, which was hosted virtually in Geneva, Switzerland on Thursday 29 April 2021, by the EMT Muhammadiyah Deployment during the COVID-19 pandemic serves as a strategy to publicise the organisation’s role and contribution in disaster management to the world.

This study showed that Muhammadiyah still experiences significant challenges and obstacles, although it employs a comprehensive response and proper strategy in handling natural hazards during the pandemic. These challenges are recognised while facing multi-hazards due to the increased complexity of personnel capacity, team logistics, and facility modifications. Other obstacles are the tracing, testing, treatment, and referral system used in assessing affected areas during the pandemic, increased risk of transmission in affected areas, and the need to suspect every patient until confirmed negative (Nihayati 2021).

Based on the study results showing these challenges and constraints, future policies and budgets should apply the principles of community-based disaster risk reduction during a pandemic. Another important suggestion is that the cooperation between governments and community organisations should be improved, especially the efforts to handle natural hazards and the COVID-19 pandemic, based on the strength of thecommunity.

## Conclusion

Muhammadiyah has been instrumental in helping the victims of natural hazards in Indonesia, both in regular times and especially during the COVID-19 outbreak. Subsequently, this study showed that natural hazards worsened the suffering of people who are already affected by the adverse effects of the COVID-19 pandemic. Muhammadiyah has played an active role by providing assistance in various regions via an appropriate strategy. Through MDMC, the organisation has followed the stages of prevention before disasters and adhered to adequate practical measures, namely disaster management methods such as mitigation, preparedness, emergency response, and recovery after a hazard during the COVID-19 pandemic.

The response and strategy used by this organisation in dealing with natural hazards during the COVID-19 pandemic, which is the study’s finding, are not only able to address the problem of this article that has not been discussed by other researchers, but can also be exemplified by other community organisations and the government. In the Muhammadiyah approach, experts understand and have practised disaster theories in many natural and non-natural management situations inside and outside Indonesia.

This study was limited to assessing Muhammadiyah’s response and strategy in managing natural hazards while the COVID-19 pandemic is still ongoing. Consequently, further comparative research should identify and map the advantages and weaknesses of actions implemented by Muhammadiyah and other organisations to facilitate mutual support and complement in helping the community overcome the multi-hazardous disasters. This is based on the idea that an organisation, no matter its size, cannot handle a hazard alone. The government cannot solely manage natural hazards without including the community. Hence, a collaboration between all parties in handling natural hazards, especially during the COVID-19 pandemic, is encouraged, as this will assist the community and lighten their burden, thereby fostering gratitude.
